# The Inflammasome and the Epidermal Growth Factor Receptor (EGFR) Are Involved in the *Staphylococcus aureus*-Mediated Induction of IL-1alpha and IL-1beta in Human Keratinocytes

**DOI:** 10.1371/journal.pone.0147118

**Published:** 2016-01-25

**Authors:** Maren Simanski, Franziska Rademacher, Lena Schröder, Regine Gläser, Jürgen Harder

**Affiliations:** Department of Dermatology, University of Kiel, Kiel, Germany; Virginia Tech University, UNITED STATES

## Abstract

*Staphylococcus* (*S*.) *aureus* is an important pathogen causing various infections including those of the skin. Keratinocytes are able to sense invading *S. aureus* and to initiate a fast defense reaction by the rapid release of innate defense mediators such as antimicrobial peptides and cytokines. There is increasing evidence that the cytokines IL-1alpha and IL-1beta, which both signal through the IL-1 receptor, play an important role in cutaneous defense against *S. aureus*. The aim of this study was to gain more insight into the underlying mechanisms leading to the *S. aureus*-induced IL-1alpha and IL-1beta expression in keratinocytes. Infection of human primary keratinocytes with *S. aureus* led to the induction of gene expression and protein secretion of IL-1alpha and IL-1beta. Full *S. aureus*-induced IL-1 protein release required the inflammasome components caspase-1 and ASC (apoptosis-associated speck-like protein containing a CARD) whereas gene induction of IL-1alpha and IL-beta by *S. aureus* was not dependent on caspase-1 and ASC. Since patients receiving anti-cancer therapy by inhibition of the epidermal growth factor receptor (EGFR) often suffer from skin infections caused by *S. aureus* we additionally evaluated whether the EGFR pathway may be involved in the IL-1alpha and IL-1beta induction by *S. aureus*. Inactivation of the EGFR with a blocking antibody decreased the *S. aureus*-mediated IL-1alpha and IL-1beta induction in primary keratinocytes. Moreover, the use of siRNA experiments revealed that ADAM17 (A Disintegrin and A Metalloprotease 17), a metalloproteinase known to mediate the shedding and release of EGFR ligands, was required for full induction of IL-1alpha and IL-1beta in keratinocytes infected with *S. aureus*. A failure of keratinocytes to adequately upregulate IL-1alpha and IL-1beta may promote *S. aureus* skin infections.

## Introduction

The Gram-positive bacterium *Staphylococcus aureus* (*S. aureus*) is a potential pathogenic bacterium responsible for a wide variety of infections. Although the nose and skin of approximately 30% of healthy people are asymptomatically colonized by *S. aureus* it is the cause of several infections ranging from minor skin infection to lethal bacteremia and sepsis [1, 2].

To adequately fend off *S. aureus* the skin has to initiate a concerted action requiring the production of defense molecules such as antimicrobial peptides and cytokines [3, 4]. Such defense molecules can be produced by keratinocytes which initiate a first rapid defense reaction upon recognition of *S. aureus*. Recognition is mediated by Toll-like receptors (TLRs) and intracellular NOD-like receptors (NLRs) which are expressed by keratinocytes and are able to detect the presence of *S. aureus* [5–7]. Antimicrobial peptides released by keratinocytes play an important role to control the growth of *S. aureus* due to their potent antimicrobial activity against *S. aureus* [8–12]. Cytokines released by keratinocytes play also an important role in cutaneous innate defense against *S. aureus* [4]. In particular IL-1alpha and IL-1beta have been reported to fulfill important tasks in the innate cutaneous defense against *S. aureus* due to their ability to promote neutrophil recruitment and to induce antimicrobial peptides and cytokines/chemokines [4, 13–16].

The aim of this study was to gain more insight into the underlying mechanisms leading to *S. aureus*-mediated induction of IL-1alpha and IL-1beta in keratinocytes. In immune cells the induced release of IL-1beta by *S. aureus* requires the inflammasome, a multi-protein complex mediating the processing and secretion of IL-1beta [17–20]. Since the role of the inflammasome in keratinocytes infected with bacteria is insufficiently explored we investigated the impact of the inflammasome on IL-1alpha and IL-1beta release in keratinocytes challenged with *S. aureus*.

There is evidence that the epidermal growth factor receptor (EGFR) plays an important role in cutaneous defense [21]. This is highlighted by the observation that patients receiving therapy by blocking the EGFR are associated with a higher risk of cutaneous infections, especially with *S. aureus* infections [22]. Given the presumable importance of IL-1 and EGFR in cutaneous defense we sought to determine whether the EGFR may be involved in the induction of IL-1 in keratinocytes infected with *S. aureus*. In addition, we also investigated the role of the metalloprotease ADAM17 (A Disintegrin and A Metalloproteinase 17) in this scenario, because ADAM17 is the principal protease required to proteolytically activate EGFR-ligands such as TGF-alpha and HB-EGF [23].

## Material and Methods

### Cell culture and stimulation

Primary foreskin-derived keratinocytes were purchased from Promocell (Heidelberg, Germany). Cells were cultured in Keratinocyte Growth Medium-2 (KGM-2, Promocell) without antibiotics at 37°C in a 5% CO_2_ atmosphere. For stimulation cells of passage 3–4 were seeded in 12-well plates and used at 1–2 days after reaching 100% confluence.

*Staphylococcus* (*S.*) *aureus* bacteria (SH 1000) were cultured in trypticase soy broth (TSB) at 37°C with shaking (200 rpm). Overnight cultures of *S. aureus* were diluted 1:50 in fresh TSB and grown for 3–4 hours until reaching an OD_600_ of 0.3–0.6. Bacteria were harvested by centrifugation, washed with Dulbecco's Phosphate-Buffered Saline (PBS, Biowest SAS, Nuaillé, France) and resuspended in KGM-2 to an OD_600_ of 0.2 corresponding to approx. 1.7 x 10^7^ bacteria ml^-1^. Approx. 5 x 10^6^ bacteria / well were centrifuged at 300 x *g* for 5 minutes onto the cells yielding a multiplicity of infection (MOI) of approximately 10. After two hours medium was removed, cells were washed with PBS and incubated for additional four hours with KGM-2 containing gentamicin (200 μg ml^-1^) to kill any remaining extracellular bacteria. Subsequently, medium was removed, centrifuged at 12.000 x *g* for 5 minutes and stored at -80°C until analyses by ELISA. The keratinocytes were washed with PBS and used for RNA isolation. In some experiments cells were treated with the caspase-1 inhibitor Ac-YVAD-CMK (Cayman, Ann Arbor, MI) or with the EGFR-blocking antibody cetuximab (Merck, Darmstadt, Germany).

### RNA isolation and cDNA synthesis

After stimulation keratinocytes were washed with PBS and cells from one well of a 12-well plate were harvested and lysed using 500 μl Crystal RNAmagic reagent (Biolab-Products, Bebensee, Germany). Total RNA was isolated according to the supplier’s protocol and resuspended in 15 μl H_2_O. RNA quantity was determined photometrically using a NanoDrop device (Peqlab, Erlangen, Germany) and 1 μg of total RNA was reversely transcribed to cDNA using oligo dT- primers and 50 Units Maxima Reverse Transcriptase (Thermo Fisher Scientific, Waltham, MA) according to the manufacturer’s protocol.

### Real-time PCR

Real-time PCR with cDNA corresponding to 10 ng total RNA as template was performed in a StepOne Real-Time PCR System (Thermo Fisher Scientific, Waltham, MA) using SYBR Premix Ex Taq II (TaKaRa Bio, Saint-Germain-en-Laye, France) as previously described [7]. The following intron spanning primers were used: IL-1alpha 5´- TGT GAC TGC CCA AGA TGA AG—3´ (forward primer) and 5´- AAG TTT GGA TGG GCA ACT GA-3´ (reverse primer); IL-1beta 5´- AAG CCC TTG CTG TAG TGG TG -3´(forward primer) and 5´- GAA GCT GAT GGC CCT AAA CA -3´(reverse primer). Primer for determination of the caspase-1, ADAM17 siRNA-mediated knockdown were purchased from Qiagen (QuantiTect Primer Assay, Cat.no. QT00001568; QT00055580 and QT00029771; Qiagen, Hilden, Germany). Serial dilutions of template cDNA were used to generate standard curves for each primer set. All quantifications were normalized to the housekeeping gene ribosomal protein L38 (RPL38) using the primer pair: 5´- TCA AGG ACT TCC TGC TCA CA -3´ (forward primer) and 5´- AAA GGT ATC TGC TGC ATC GAA -3´ (reverse primer). Relative expression is given as a ratio between target gene expression and RPL38 expression.

### siRNA experiments

SilencerSelect siRNAs specific for caspase-1 (s2408), ASC (s195168), ADAM17 (s13720), NLRP3 (s141555) and a non-silencing control siRNA (4390844) were purchased from Life Technologies. Primary keratinocytes were cultured in 12-well plates and transfected with 5–10 nM siRNA at 40–60% confluence using 2 μl of transfection reagent HiPerFect (Qiagen, Hilden, Germany). After 16–20 hours, siRNA was removed and cells were further cultured for 2–3 days until they reached 100% confluence for stimulation with *S. aureus*. Real-time PCR analyses were used to determine the reduction of gene expression in the cells transfected with specific siRNA as compared with the cells transfected with a non-silencing control siRNA. Reduction of specific siRNA-mediated gene expression was always more than 60% (S1 Fig).

### ELISA

Secreted protein levels of IL-1alpha and IL-1beta were determined by measuring protein contents in 100 μl cell culture supernatants using ELISA. ELISA kits specific for IL-1alpha, IL-1beta, pro-IL-1beta and p20 subunit of caspase-1 were purchased from R&D Systems (Minneapolis, MN). ELISA was performed in 96-well plates (MaxiSorp; Thermo Fisher Scientific) according to the manufacturer's protocol. The detection limit of the IL-1alpha and IL-1beta ELISA was 3.4–7.8 pg/ml, the detection limit of the caspase-1 and pro-IL-1beta ELISA was at 6.2 pg/ml and 11.6 pg/ml, respectively.

### Analysis of caspase-1 activation

To analyze caspase-1 activation we transfected the primary keratinocytes 48 h prior *S. aureus* stimulation with an iGLuc plasmid (kindly provided by Prof. Hornung, Bonn, Germany) using the transfection reagent FuGENE HD (Promega, Mannheim, Germany). This iGLuc luciferase-based plasmid serves as a specific and sensitive caspase-1 reporter based on the biological activity of a pro–IL-1beta–*Gaussia* luciferase (iGLuc) fusion construct. Proteolytic activity of caspase-1 can be monitored by analysis of luciferase activity in the cell supernatant [24]. To measure iGLuc-based luciferase activity we mixed 50 μl cell supernatant with 50 μl of the luciferase substrate coelenterazine (4.4 μM diluted in water) and measured luciferase activity using a TD-20/20 luminometer (Turner Design, Sunnyvale, CA).

### Statistical Analysis

Statistical differences were assessed using two-tailed Student`s *t*-test. A p-value ≤ 0.05 was considered significant.

## Results

### *S. aureus* induces gene and protein expression of IL-1alpha and IL-1beta in primary keratinocytes

**Fig 1:**
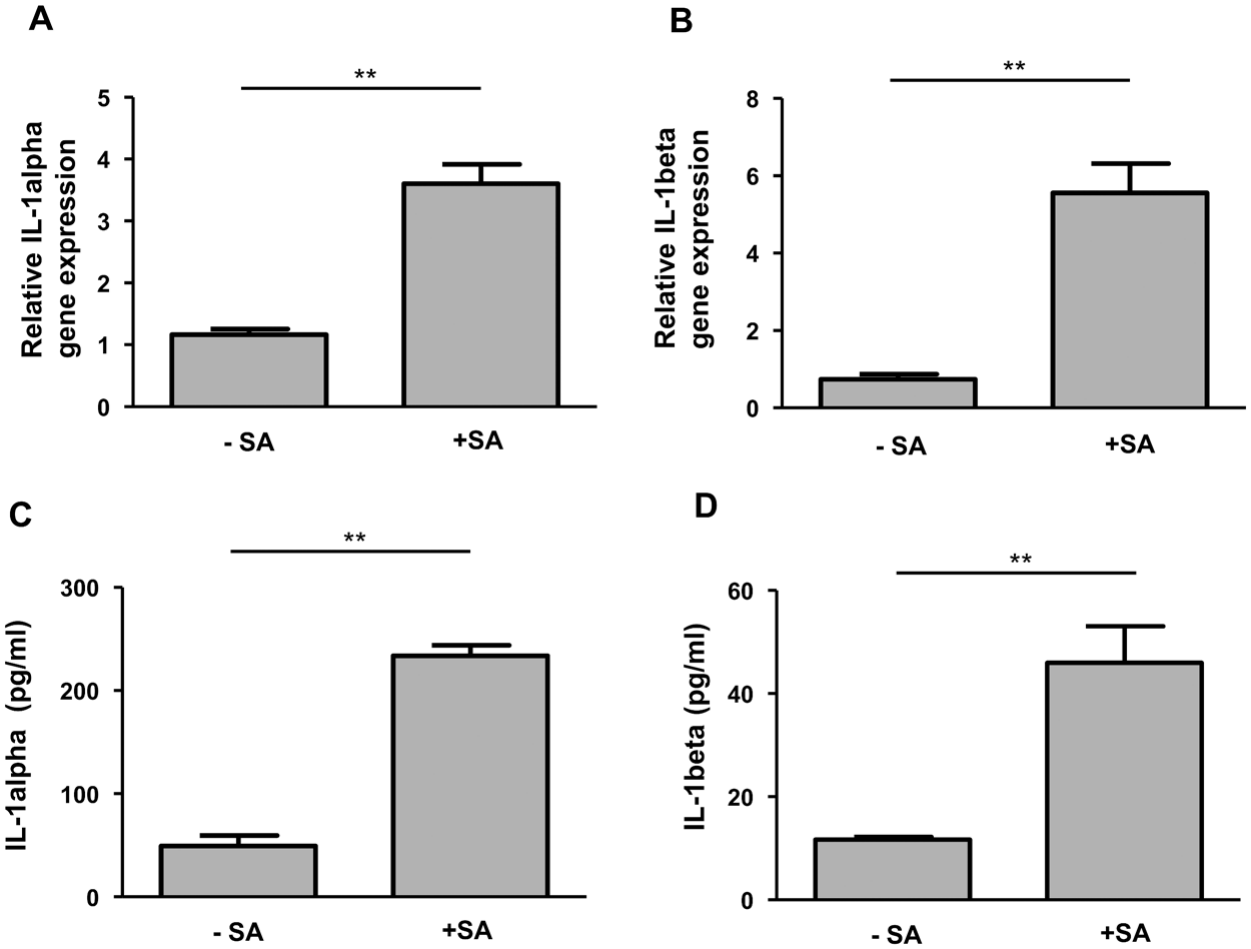
Infection of primary keratinocytes with *S. aureus* induces expression of IL-1alpha and IL-1beta. Human primary keratinocytes were left unstimulated (- SA) or were stimulated with *S. aureus* (+ SA) for a total of six hours. Gene expression of IL-1alpha (A) and IL-1beta (B) was analysed by real-time PCR. Protein secretion of IL-1alpha (C) and IL-1beta (D) was measured by ELISA. Data are means ± SEM (**p< 0.01, Student`s *t*-test, n = 3).

Stimulation of primary keratinocytes with living *S. aureus* resulted in an increased IL-1alpha and IL-1beta gene expression as measured by real-time PCR (Fig 1A and 1B). In line with these results *S. aureus* also induced the secretion of IL-1alpha and IL-1beta in primary keratinocytes as measured by ELISA (Fig 1C and 1D).

### Caspase-1 is involved in the *S. aureus*-induced release of IL-1alpha and IL-1beta

**Fig 2:**
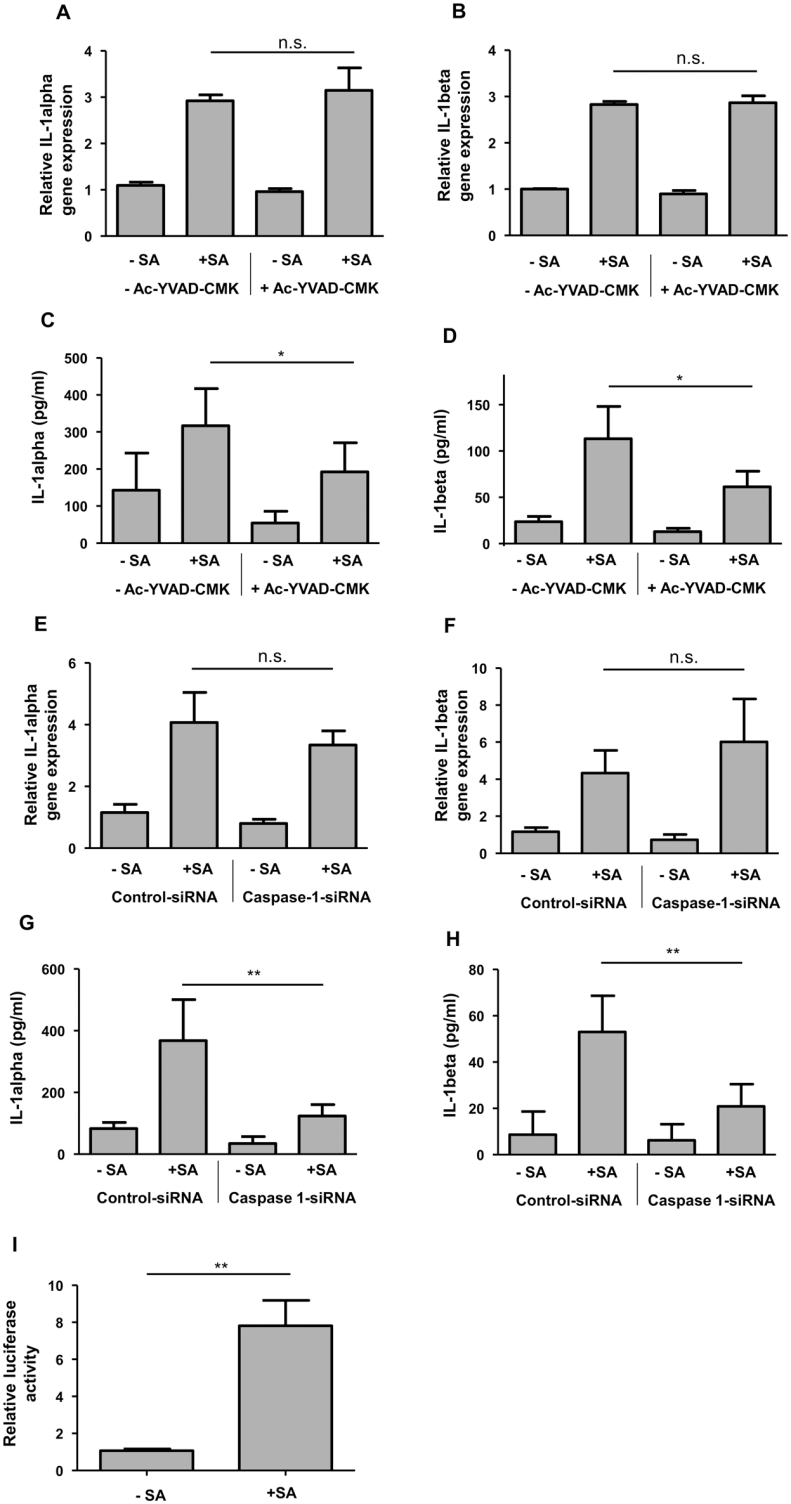
Caspase-1 is involved in the *S. aureus*-induced IL-1alpha and IL-1beta release in keratinocytes. Primary keratinocytes were left unstimulated (- SA) or were stimulated with *S. aureus* (+ SA) for a total of six hours. To investigate the influence of caspase-1 on IL-1alpha and IL-1beta expression the cells were co-treated with a caspase-1 inhibitor (Ac-YVAD-CMK, 30 μM). Gene expression of IL-1alpha (A) and IL-1beta (B) was measured by real-time PCR. Protein secretion of IL-1alpha (C) and IL-1beta (D) was determined by ELISA. To further assess the influence of caspase-1, cells were transfected with a caspase-1 specific siRNA or a non-silencing control siRNA. Gene expression of IL-1alpha (E) and IL-1beta (F) was measured by real-time PCR, protein secretion of IL-1alpha (G) and IL-1beta (H) was analysed by ELISA. To directly assess activation of caspase-1 cells were transfected with a caspase-1 luciferase reporter plasmid and proteolytic activity of caspase-1 was determined by analysis of luciferase activity (I). Data are means ± SEM (*p< 0.05, **p< 0.01, n.s. = not significant, Student`s *t*-test, n = 6 (A-H) and n = 5 (I)).

Since caspase-1 is the central protease of the inflammasome known to mediate cleavage and secretion of IL-1beta we analyzed the influence of caspase-1 on the *S. aureus*-mediated IL-1alpha and IL-1beta release. To this end primary keratinocytes were stimulated with *S. aureus* in the presence or absence (only DMSO as vehicle control) of the caspase-1 inhibitor Ac-YVAD-CMK (30 μM). This resulted in a diminished secretion of both proteins indicating that caspase-1 is required for full secretion of IL-1alpha and IL-1beta in keratinocytes infected with *S. aureus* (Fig 2C and 2D). In contrast, *S. aureus*-mediated gene expression of IL-1alpha and IL-1beta was not affected by the caspase-1 inhibitor (Fig 2A and 2B).

To further evaluate the role of caspase-1 primary keratinocytes were transfected with a caspase-1 specific siRNA or a non-silencing control siRNA. Keratinocytes treated with the caspase-1 siRNA showed a diminished protein release of IL-1alpha and IL-1beta (Fig 2G and 2H). In contrast, gene expression of IL-1alpha and IL-1beta was not significantly altered in the cells with siRNA-mediated reduced caspase-1 expression (Fig 2E and 2F).

To determine direct activation of caspase-1 by *S. aureus* keratinocytes were transfected with a caspase-1 luciferase reporter plasmid (iGLuc). Induction of luciferase activity upon *S. aureus* treatment indicated activation of caspase-1 proteolytic activity (Fig 2I)

Activation of caspase-1 was further confirmed by measuring the presence of caspase-1 p20 subunits secreted into the supernatants of the keratinocytes stimulated with *S. aureus*. Increased secretion of caspase-1 upon treatment with *S. aureus* indicated activation of caspase-1 (S2 Fig).

### ASC is involved in the *S. aureus*-mediated release of IL-1alpha and IL-1beta

**Fig 3:**
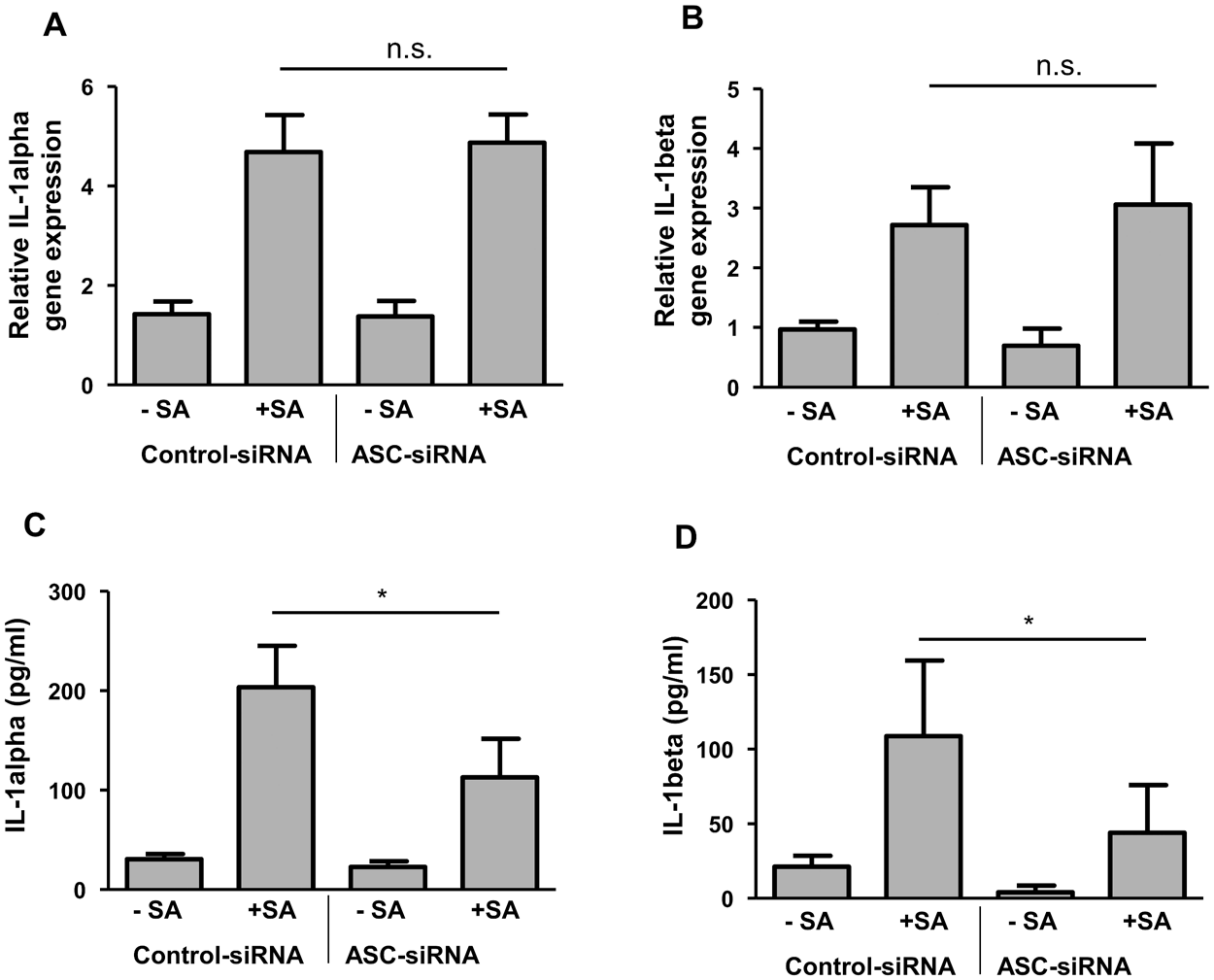
ASC is involved in the *S. aureus*-induced IL-1alpha and IL-1beta release in keratinocytes. Primary keratinocytes were left unstimulated (- SA) or were stimulated with *S. aureus* (+ SA) for a total of six hours. To investigate the influence of ASC on the IL-1alpha and IL-1beta expression the cells were transfected with an ASC specific siRNA or a non-silencing control siRNA. Gene expression of IL-1alpha (A) and IL-1beta (B) was measured by real-time PCR, protein secretion of IL-1alpha (C) and IL-1beta (D) was analysed by ELISA. Data are means ± SEM (*p< 0.05, n.s. = not significant, Student`s *t*-test, n = 6).

To assess the influence of the inflammasome adaptor molecule ASC (apoptosis-associated speck-like protein containing a CARD) we transfected primary keratinocytes with an ASC specific siRNA or a non-silencing control siRNA. Reduced ASC expression correlated with a reduced secretion of IL-1alpha and IL-1beta (Fig 3C and 3D). In contrast, gene expression of IL-1alpha and IL-1beta was not affected (Fig 3A and 3B) indicating that ASC participates in the *S. aureus*-induced protein release but does not influence the *S. aureus*-induced gene expression of IL-1alpha and IL-1beta. Release of IL-1alpha and IL-1beta was not affected in *S. aureus*-stimulated keratinocytes treated with specific siRNA targeting the inflammasome-derived component NLRP3 (S3 Fig).

### Epidermal growth factor receptor (EGFR) is involved in the *S. aureus*-induced expression of IL-1alpha and IL-1beta

**Fig 4:**
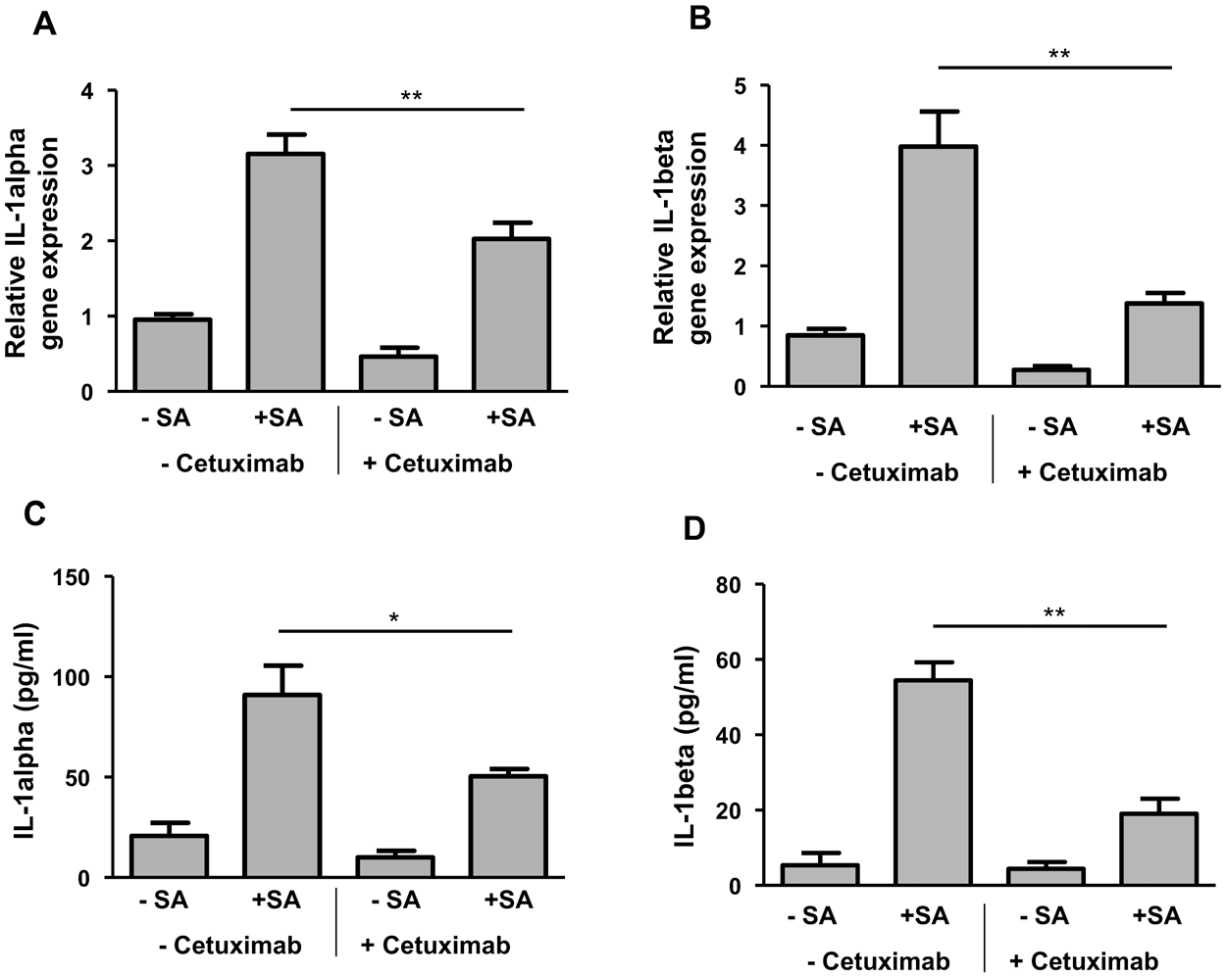
EGFR is involved in the *S. aureus*-induced IL-1alpha and IL-1beta release in keratinocytes. Primary keratinocytes were left unstimulated (- SA) or were stimulated with *S. aureus* (+ SA) for a total of six hours. To investigate the influence of EGFR on IL-1alpha and IL-1beta expression the cells were co-treated with or without the EGFR blocking antibody cetuximab. Gene expression of IL-1alpha (A) and IL-1beta (B) was determined by real-time PCR. Protein secretion of IL-1alpha (C) and IL-1beta (D) was analysed by ELISA. Data are means ± SEM (**p< 0.01, Student`s *t*-test, n = 9).

To investigate the role of the EGFR-pathway for the *S. aureus*-induced expression of IL-1alpha and IL-1beta we treated the keratinocytes with the EGFR-blocking antibody cetuximab. The cells were pre-incubated for 1h with 20 μg/ml cetuximab and further co-treated with 20 μg/ml cetuximab during stimulation with *S. aureus*. IL-alpha and IL-1beta gene induction by *S. aureus* in primary keratinocytes was decreased by incubation with cetuximab (Fig 4A and 4B). Parallel with diminished gene induction the *S. aureus*-induced protein secretion of IL-1alpha and IL-1beta was reduced when the EGFR was blocked by cetuximab (Fig 4C and 4D). These results indicate that full induction of IL-1alpha and IL-1beta in keratinocytes infected with *S. aureus* requires the EGFR.

### ADAM17 is involved in the *S. aureus*-induced expression of IL-1alpha and IL-1beta

**Fig 5:**
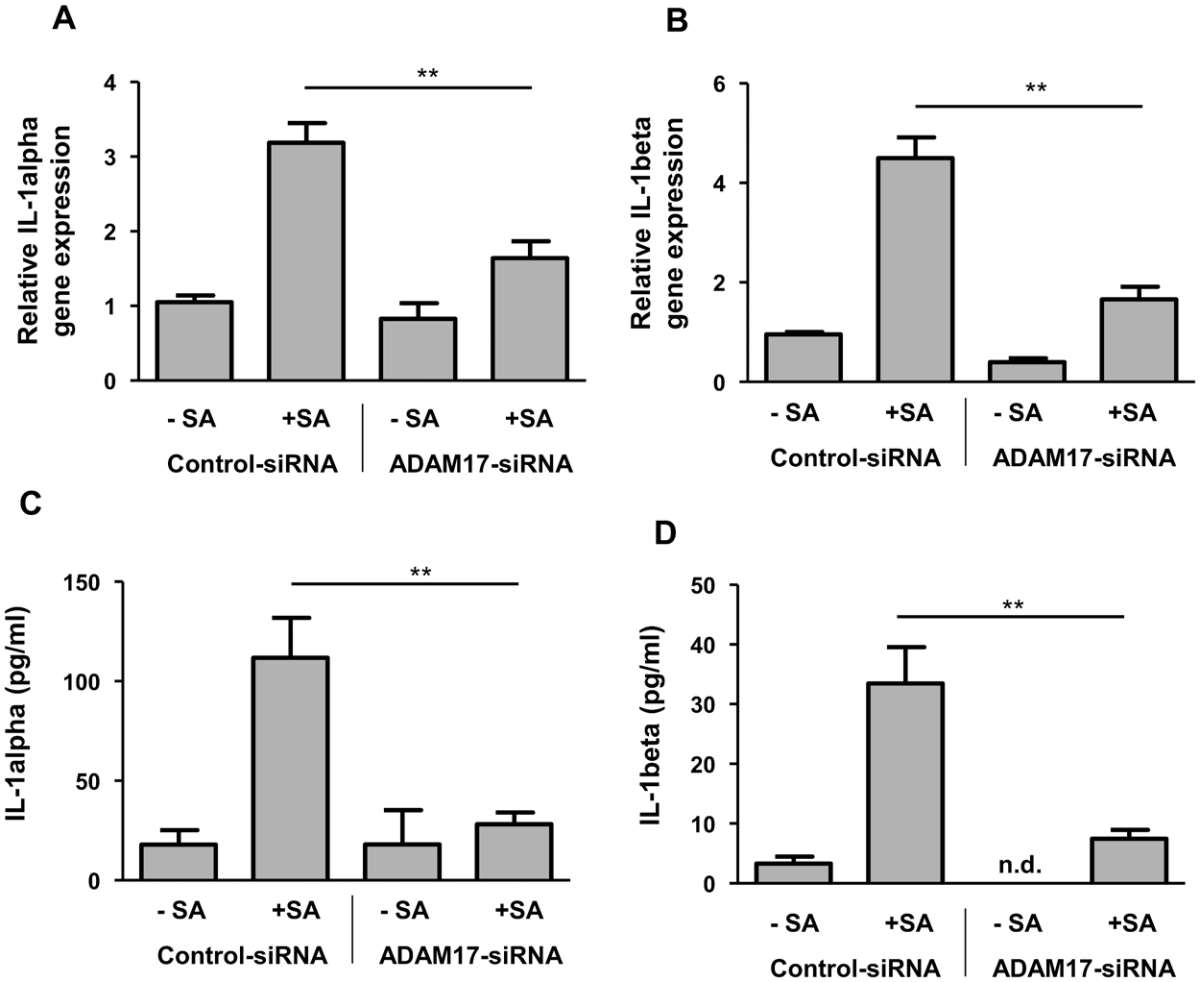
ADAM17 is involved in the *S. aureus*-induced IL-1alpha and IL-1beta release in keratinocytes. Primary keratinocytes were left unstimulated (- SA) or were stimulated with *S. aureus* (+ SA) for a total of six hours. To investigate the influence of ADAM17 on the IL-1alpha and IL-1beta secretion the cells were transfected with an ADAM17 specific siRNA or a non-silencing control siRNA. Gene expression of IL-1alpha (A) and IL-1beta (B) was measured by real-time PCR, protein secretion of IL-1alpha (C) and IL-1beta (D) was analysed by ELISA. Data are means ± SEM (**p< 0.01, Student`s *t*-test, n = 6, n.d. = not determined (< 3.4 pg/ml)).

To analyze the role of ADAM17 for the *S. aureus*-induced expression of IL-1alpha and IL-1beta we transfected primary keratinocytes with an ADAM17 specific siRNA or a non-silencing control siRNA. Reduced ADAM17 expression correlated with a reduced *S. aureus*-mediated gene induction of IL-1alpha and IL-1beta (Fig 5A and 5B). Similarly, protein release of IL-1alpha and IL-1beta was decreased in the keratinocytes treated with the ADAM17-siRNA (Fig 5C and 5D). These data indicate that ADAM17 is involved in the *S. aureus*-induced gene and protein expression of IL-1alpha and IL-1beta.

## Discussion

Our data show that keratinocytes infected with *S. aureus* have the capacity to rapidly secrete IL-1alpha and IL-1beta. Since these cytokines are important for activation and recruitment of effector cells such as neutrophils [4] it is likely that release of IL-1 by keratinocytes is an important first step in innate cutaneous defense to hamper the spread of *S. aureus*. In mice studies it has been reported that mice deficient in IL-1beta showed impaired bacterial clearance and defective neutrophil recruitment after intradermal *S. aureus* infection [18]. This highlights the general importance of IL-1beta in defense against *S. aureus*. In that study mice deficient in IL-1alpha revealed a similar phenotype than wildtype mice suggesting that IL-1alpha plays no major role in these experimental settings [18]. However, since *S. aureus* were subcutaneously injected into the mice thereby bypassing the epidermal barrier it is reasonable that keratinocytes and keratinocytes-derived IL-1 were not involved in this scenario.

The levels of IL-1alpha released by our *S. aureus*-infected keratinocytes were generally higher than the IL-1beta levels. This is in concordance with previous studies reporting high levels of IL-1alpha in healthy epidermis. An IL-1alpha to IL-1beta ratio of 40:1 has been reported in normal epidermis [25]. The importance of IL-1alpha in cutaneous defense has been documented in several studies. For example, it has been reported that IL-1alpha induces the expression of antimicrobial peptides in keratinocytes and increases the antibacterial activity of keratinocytes and an epidermal skin equivalent [26]. A strong synergism of IL-1alpha with EGFR ligands resulted in a potent induction of antimicrobial peptides and chemokines [27]. Furthermore, it has been shown that the induction of chemokines in keratinocytes stimulated with heat-killed *S. aureus* was dependent on endocrine IL-1alpha (but not IL-1beta) [15]. Another study described that the release of IL-1alpha in keratinocytes infected with herpes simplex virus-1 (HSV-1) was important for recruitment of immune cells to the epidermis during the very early stages of skin infection thereby providing protection against the spread of HSV-1 [14]. Taken together these studies indicate that IL-1alpha released by keratinocytes may play an important role in epidermal defense.

The induced protein secretion of IL-1alpha and IL-1beta by the keratinocytes infected with *S. aureus* was—at least partially—dependent on the inflammasome components caspase-1 and ASC. In contrast to protein secretion, *S. aureus*-mediated gene induction of IL-1alpha and IL-1beta was not influenced by caspase-1 and ASC indicating that caspase-1 and ASC mediate induced secretion but not induced gene expression of IL-1alpha and IL-1beta in *S. aureus*-infected keratinocytes. For IL-1beta the caspase-1- and ASC-dependent secretion is somewhat expectable as release of IL-1beta in various cell types by different stimuli requires activation of the inflammasome leading to the caspase-1 mediated cleavage of pro-IL-1beta and secretion of mature IL-1beta [28]. Although there is experimental evidence that our used IL-1beta ELISA is detecting primarily the cleaved form of IL-1beta [29] we also analysed the released IL-1beta with an ELISA that specifically detects the full-length form of IL-1beta (pro-IL1beta). Using this ELISA we failed to detect any pro-IL-1beta (detection limit 11.6 pg/ml, data not shown) indicating that the released IL-1beta of the keratinocytes stimulated with *S. aureus* represents predominantly the cleaved form. For bone-marrow derived mouse immune cells stimulated with *S. aureus* components an inflammasome-dependent secretion of IL-1beta has been well documented [17–20]. In addition, Soong et al. reported a caspase-1 dependent IL-1beta release in the keratinocytes cell line HaCaT treated with *S. aureus* [30]. Thus, our results confirm that *S. aureus* is able to activate the inflammasome in keratinocytes leading to the increased secretion of cleaved IL-1beta. Several studies reported that the *S. aureus*-mediated secretion of IL-1beta in bone-marrow derived mouse immune cells required the inflammasome protein NLRP3 [17, 19, 20]. However, we observed that keratinocytes with a siRNA-mediated decrease of NLRP3 expression released similar amounts of IL-1beta as well as IL-1alpha after stimulation with *S. aureus* indicating that NLRP3 plays no role in this scenario (S3 Fig).

Our data indicate that the *S. aureus*-mediated IL-1alpha secretion requires also the inflammasome. Although it is well known that the inflammasome-associated enzymatic activity of caspase-1 is responsible for the processing and release of IL-1beta in various cell types, the role of caspase-1 for the IL-1alpha secretion is less explored. We do not know whether *S. aureus* induced the secretion of the full-length form or processed form of IL-1alpha but it is likely that in our scenario caspase-1 does not play a role in processing IL-1alpha because, in contrast to IL-1beta, caspase-1 does not cleave IL-1alpha [31]. In addition, IL-1alpha is also biologically active in its intact full-length form [32–34] and processed IL-1alpha as well as pro-IL-1alpha bind to the IL-1receptor IL-1R1 [33]. However, other proteases such as calpain and granzyme B, which both are expressed in keratinocytes, are able to process IL-1alpha [35] and it has been reported that IL-1alpha processed by these enzymes shows enhanced biological activity [36]. This is in contrast to other studies demonstrating that the processed and unprocessed forms of IL-1alpha have a similar degree of activity [29, 32]. Together, these reports suggest that the IL-1alpha released in the *S. aureus*-treated keratinocytes exhibit biological activity. If the IL-1alpha released by the *S. aureus*-treated keratinocytes represents primarily the full-length or processed form and if other proteases might play a role in this scenario and if this has an impact on the biological activity of the released IL-1alpha remains to be shown in future experiments.

As mentioned above caspase-1 does not cleave IL-1alpha. Nevertheless, there is increasing evidence that in specific scenarios caspase-1 and an active inflammasome are required for IL-1alpha release [34]. In keratinocytes it has been shown that nanoparticles (nano-TiO2) and the ionophore nigericin induce the ASC-dependent secretion of IL-1alpha [37]. Moreover, it has been reported that caspase-1 is required for the unconventional secretion of pro-IL-1alpha in UV-treated keratinocytes. Although pro-IL-1alpha is not a substrate for caspase-1, the caspase-1-dependent release of pro-IL-1apha required enzymatically active caspase-1 and was accompanied by a physical interaction of pro-IL-1alpha with caspase-1 [38]. However, it is not clear if the parallel secreted caspase-1 served as a carrier to release IL-1alpha or if caspase-1 activated a secretion machinery via its activity [38]. In line with the above mentioned studies our study highlights another scenario where the inflammasome is required for the induced release of IL-1alpha in keratinocytes.

Recently, it has been reported that caspase-4 physically interacts with caspase-1 and that active caspase-4 is required for IL-1beta secretion by keratinocytes irradiated with UVB [39]. Furthermore, activation of TLR-3 in keratinocytes by poly I:C induced a caspase-4 dependent release of IL-1beta [40]. In addition, caspase-8 has also been implicated in processing of IL-1beta [41–43]. Thus, it remains to be shown whether caspase- 4, caspase-8 and probably other proteases may play also a role in the *S. aureus*-mediated secretion of IL-1alpha and IL-1beta by keratinocytes.

We have shown that an appropriate induction of IL-1alpha and IL-1beta by keratinocytes infected with *S. aureus* requires activation of the EGFR. It has been reported that patients with anti-EGFR therapy often suffer from cutaneous infection caused by *S. aureus* [21, 22]. Thus, it is reasonable to hypothesize that a failure of *S. aureus*-mediated IL-1 induction in patients receiving anti-EGFR therapy may contribute to the increased *S. aureus* infection rate seen in these patients.

The metalloprotease ADAM17 (A Disintegrin and A Metalloproteinase 17), also known as TACE (tumor necrosis factor-α-converting enzyme), has been implicated into the upstream EGFR signal transduction pathway because ADAM17 is responsible for the cleavage and subsequent release (shedding) of several EGFR ligands such as TGF-alpha, amphiregulin and HB-EGF [23]. Our observed ADAM17-dependent IL-1 induction by *S. aureus* gives rise to the hypothesis that *S. aureus* may activate ADAM17 leading to the shedding of EGFR ligands which in turn activate the EGFR. In line with such hypothesis it has been reported that the *S. aureus* toxic shock syndrome toxin-1 (TSST-1) activated ADAM17 in vaginal epithelial cells. ADAM17 then cleaved EGFR ligands leading to EGFR activation and subsequent mitogen-activated protein kinase-mediated IL-8 secretion [44]. In contrast to this ADAM17-mediated autocrine activation of EGFR by released EGFR ligands it has also been reported that the protein A of *S. aureus* was able to directly stimulate phosphorylation of the EGFR. This protein A-mediated activation of the EGFR led to the subsequent phosphorylation and activation of ADAM17 [45]. In the case of our study it remains to be shown whether *S. aureus* first activates the EGFR or ADAM17.

It has been reported that the skin of two children with a loss-of-function mutation in ADAM17 was prone to infection with *S. aureus* [46]. This observation further strengthens the hypothesis that ADAM17 has an important function in epithelial defense against *S. aureus*. To which extend the ADAM17-mediated induction of IL-1alpha and IL-1beta may play a role in such scenario remains to be shown.

Until now it is unclear which *S. aureus*-derived factor(s) may be responsible for the observed induction of IL-1alpha and IL-1beta in keratinocytes infected with *S. aureus*. It has been reported that the alpha-toxin (alpha-hemolysin) of *S. aureus* may mediate EGFR-dependent proliferation in keratinocytes [47]. In addition, the alpha- and beta-hemolysins in combination with lipoproteins from *S. aureus* have also been reported to activate the inflammasome in mouse macrophages [19]. Another study which also used mouse macrophages infected with *S. aureus* showed that lysozyme-dependent degradation of peptidoglycan within phagolysosomes was necessary to induce IL-1beta secretion [20]. In airway epithelial cells it has been documented that protein A derived from *S. aureus* was able to activate phosphorylation of EGFR and subsequent ADAM17 activation [45]. Future studies have to figure out which *S. aureus*-derived factors are involved in the observed IL-1alpha and IL-1beta induction in keratinocytes.

Taken together our study highlights the importance of the inflammasome and the activation of the EGFR pathway for the induction and release of IL-1alpha and IL-1beta in keratinocytes infected with *S. aureus*. Future studies have to analyze the potential contribution of other pathways in this scenario and whether a complete shutdown of the EGFR pathway and the inflammasome would result in a complete abolishment of the *S. aureus*-mediated IL-1alpha and IL-1beta induction in keratinocytes. A failure to adequately induce and release keratinocytes-derived IL-1 may facilitate skin infections.

## Supporting Information

S1 Fig Knockdown efficiency of siRNA treatment. To analyze the effect of the siRNA treatment relative changes in gene expression of keratinocytes treated with siRNA for caspase-1 (A), ASC (B), ADAM17 (C) and NLRP3 (D) were determined by real-time PCR. Data are means ± SEM (**p< 0.01, Student`s *t*-test, n = 6).
(PDF)

S2 Fig Secretion of caspase-1. Primary keratinocytes were left unstimulated (- SA) or were stimulated with *S. aureus* (+ SA) for a total of six hours. A**c**tivation of caspase-1 was determined by ELISA of the caspase-1 p20 subunit secreted in the cell culture supernatant. For control purposes gene expression of caspase-1 downregulated by capsase-1 specific siRNA is also shown. Data are means ± SEM (**p< 0.01, Student`s *t*-test, n = 3).
(PDF)

S3 Fig Knockdown of NLRP3 does not influence *S. aureus*-induced IL-1alpha and IL-1beta release. Primary keratinocytes were left unstimulated (- SA) or were stimulated with *S. aureus* (+ SA) for a total of six hours. To investigate the influence of NLRP3 on the IL-1alpha and IL-1beta release the cells were transfected with an NLRP3 specific siRNA or a non-silencing control siRNA. Protein secretion of IL-1alpha (A) and IL-1beta (B) was analysed by ELISA. Data are means ± SEM (n.s. = not significant, Student`s t-test, n = 6).
(PDF)
