# Supplementary figures and images for: The Inflammasome and the Epidermal Growth Factor Receptor (EGFR) Are Involved in the Staphylococcus aureus-Mediated Induction of IL-1alpha and IL-1beta in Human Keratinocytes
Source: PLoS One. 2016 Jan 25;11(1):e0147118. doi: 10.1371/journal.pone.0147118 (PMC4726826; doi:10.1371/journal.pone.0147118)

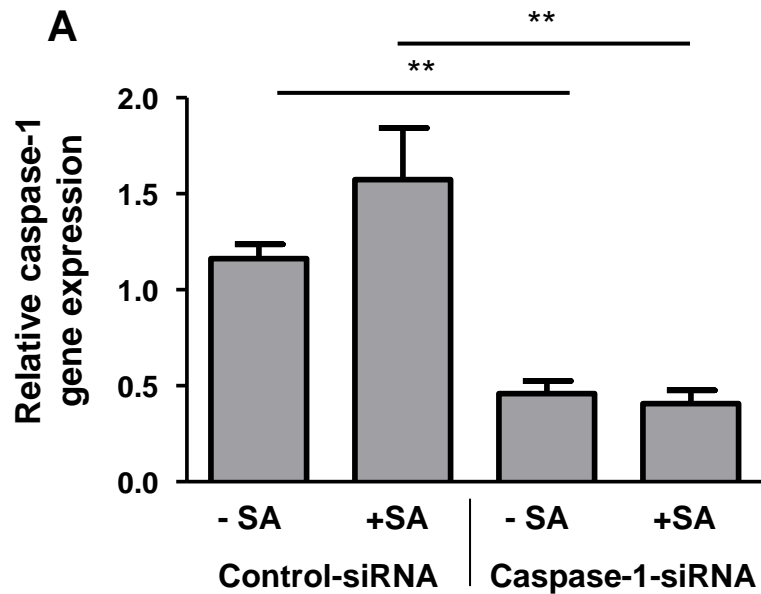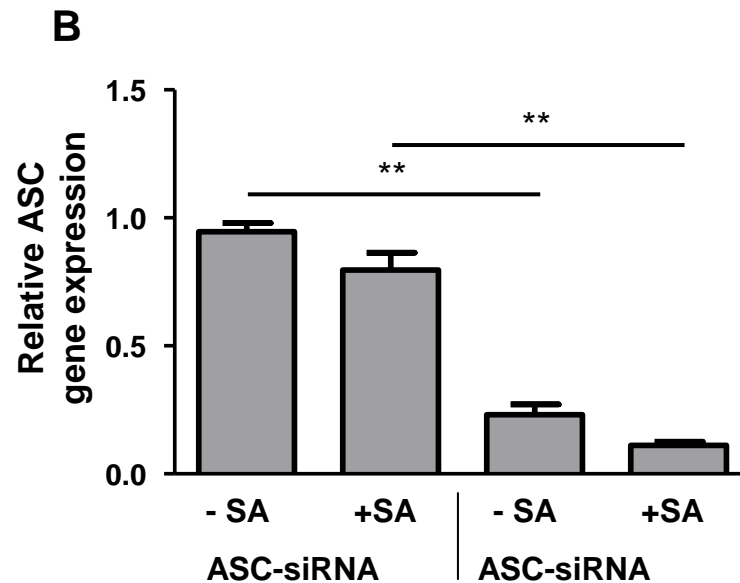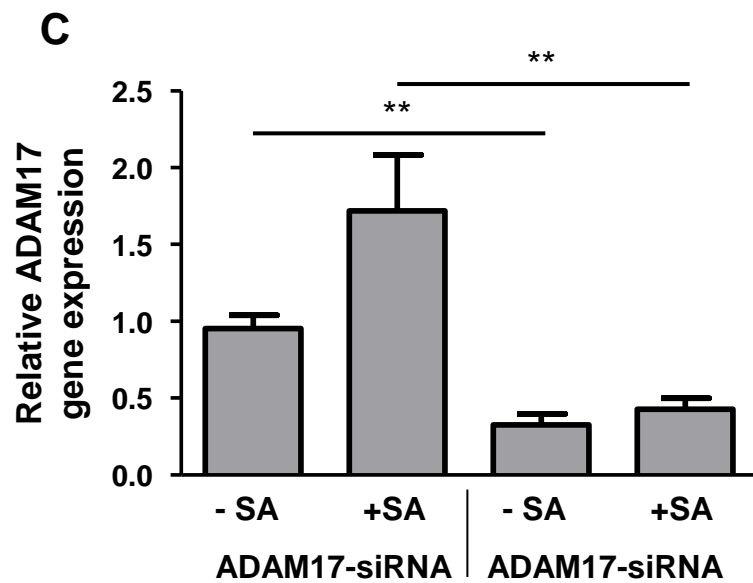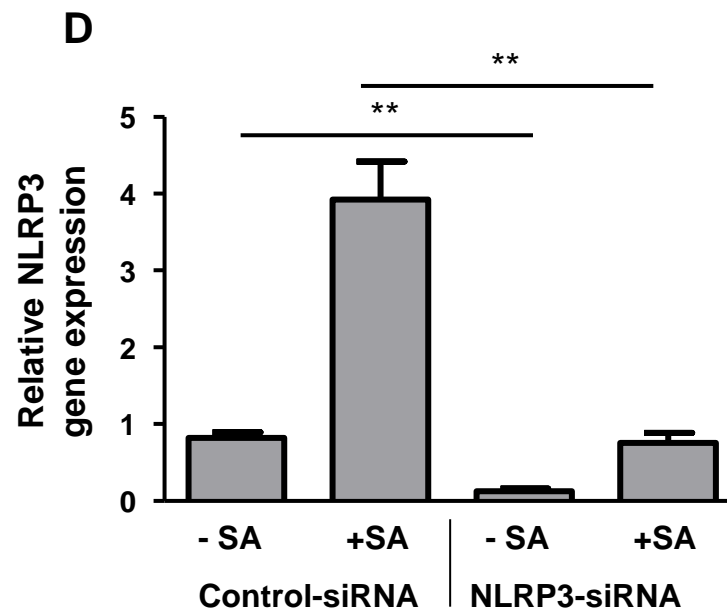

Supplement: S1 Fig — To analyze the effect of the siRNA treatment relative changes in gene expression of keratinocytes treated with siRNA for caspase-1 (A), ASC (B), ADAM17 (C) and NLRP3 (D) were determined by real-time PCR. Data are means ± SEM (**p< 0.01, Student`s t-test, n = 6). (PDF) [file pone.0147118.s001.pdf]

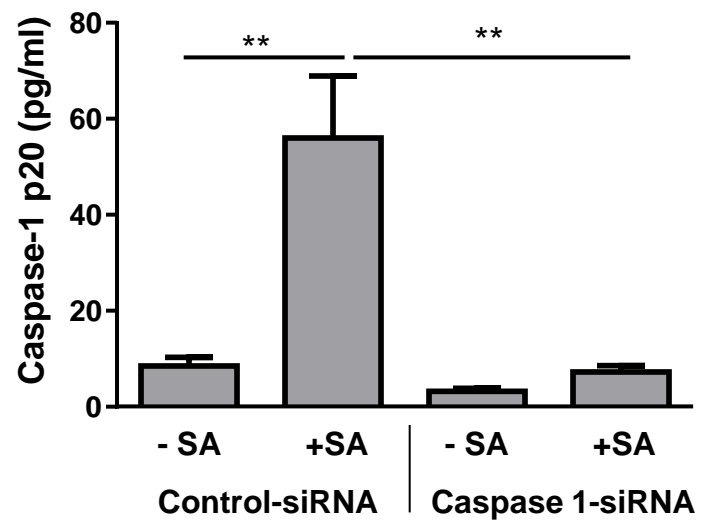

Supplement: S2 Fig — Primary keratinocytes were left unstimulated (- SA) or were stimulated with S. aureus (+ SA) for a total of six hours. Activation of caspase-1 was determined by ELISA of the caspase-1 p20 subunit secreted in the cell culture supernatant. For control purposes gene expression of caspase-1 downregulated by capsase-1 specific siRNA is also shown. Data are means ± SEM (**p< 0.01, Student`s t-test, n = 3). (PDF) [file pone.0147118.s002.pdf]

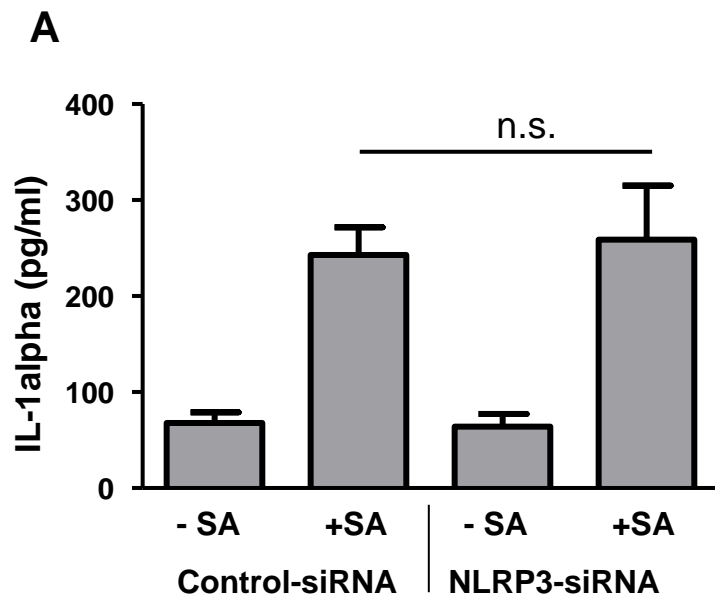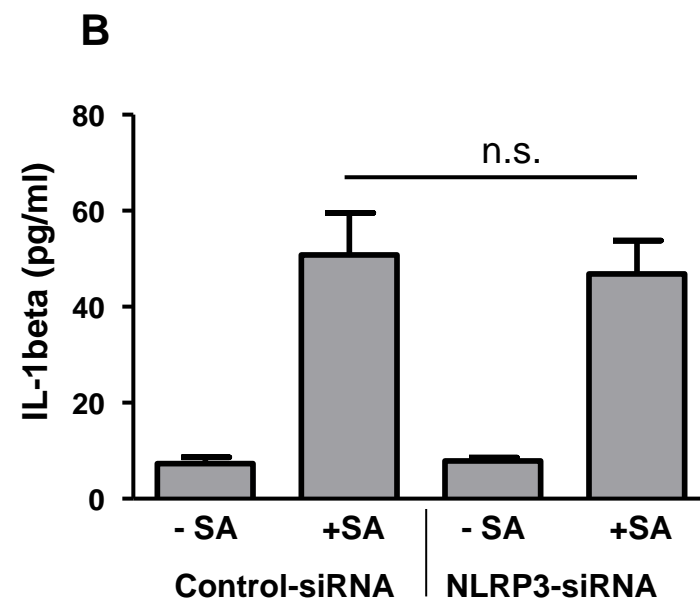

Supplement: S3 Fig — Primary keratinocytes were left unstimulated (- SA) or were stimulated with S. aureus (+ SA) for a total of six hours. To investigate the influence of NLRP3 on the IL-1alpha and IL-1beta release the cells were transfected with an NLRP3 specific siRNA or a non-silencing control siRNA. Protein secretion of IL-1alpha (A) and IL-1beta (B) was analysed by ELISA. Data are means ± SEM (n.s. = not significant, Student`s t-test, n = 6). (PDF) [file pone.0147118.s003.pdf]
